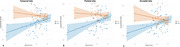# Glymphatic dysfunction and β‐amyloid deposition in patients with idiopathic normal pressure hydrocephalus

**DOI:** 10.1002/alz70856_105376

**Published:** 2026-01-07

**Authors:** Hanlin Cai, Ruihan Wang, Li Li, Keru Huang, Hui Gao, Feng Yang, Shiyu Feng, Linyuan Qin, Xiyue Yang, Shan Wang, Qian Liao, Yi Liu, Dong Zhou, Jiaojiang He, Liangxue Zhou, Rong Tian, Na Hu, Qin Chen

**Affiliations:** ^1^ West China Hospital of Sichuan University, Chengdu, Sichuan, China

## Abstract

**Background:**

Idiopathic normal pressure hydrocephalus (iNPH) often co‐occurs with beta‐amyloid deposition, both of which share a common pathophysiology involving glymphatic dysfunction. This study aimed to investigate the clinical impact of beta‐amyloid co‐pathology and the effects of glymphatic dysfunction on beta‐amyloid deposition in patients with iNPH.

**Methods:**

Patients diagnosed with probable iNPH (*n* = 60; 28 with A+, positive amyloid PET; 32 with A‐, negative amyloid PET) and Alzheimer's disease (AD) (*n* = 30, A+T+N+) were enrolled from prospective cohorts at the West China Hospital of Sichuan University. All participants underwent neuropsychological tests, magnetic resonance imaging and ^18^F‐AV45 PET. The choroid plexus volume/estimated total intracranial volume (CPV/eTIV) and ^18^F‐AV45 PET standard uptake value ratio (SUVR) were calculated based on automatic segmentation to evaluate the glymphatic function and amyloid burden. ANOVA and Kruskal‐Wallis test were used for group comparison between iNPH with positive amyloid PET and other groups, and Bonferroni correction was used for post‐hoc analysis. Multivariate generalized linear models were constructed to analyze the association between CPV/eTIV and ^18^F‐AV45 PET SUVR in patients with iNPH and AD.

**Result:**

Patients with iNPH A+ exhibited the highest CPV/eTIV compared to the patients with iNPH A‐ (*p* = 0.006). Patients with iNPH A+ showed lower MMSE score compared to iNPH A‐ (P_Bon_=0.013) and AD (P_Bon_=0.039), while no significant difference was found between iNPH A‐ and AD. In iNPH patients, higher CPV/eTIV were associated with higher ^18^F‐AV45 PET SUVR in temporal (*p* = 0.007), parietal (*p* = 0.002), and occipital lobes (*p* = 0.004); however, no association was observed in patients with AD in these regions.

**Conclusion:**

Our study demonstrated that beta‐amyloid co‐pathology may exacerbate the cognitive symptoms of iNPH. Moreover, glymphatic dysfunction might play a distinct role in promoting beta‐amyloid deposition in iNPH compared to Alzheimer's disease.